# Motor Development in Children with Cerebral Palsy in Sweden—A Population-Based Longitudinal Register Study

**DOI:** 10.3390/children10121864

**Published:** 2023-11-28

**Authors:** Cecilia Lidbeck, Henrike Häbel, Caroline Martinsson, Katina Pettersson, Kristina Löwing

**Affiliations:** 1 Division of Paediatric Neurology, Department of Women’s and Children’s Health, Karolinska Institutet, 171 76 Stockholm, Sweden; kristina.lowing@ki.se; 2 Astrid Lindgren Childrens Hospital, Karolinska University Hospital, 171 76 Stockholm, Sweden; 3 Medical Statistics Unit, Department of Learning, Informatics, Management and Ethics, Karolinska Institutet, 171 77 Stockholm, Sweden; henrike.habel@ki.se; 4 Independent Researcher, 507 60 Borås, Sweden; caroline.martinsson@cpup.se; 5 Centre for Clinical Research, Uppsala University—Region Västmanland, 721 89 Västerås, Sweden; katina.pettersson@regionvastmanland.se

**Keywords:** cerebral palsy, children, gross motor function classification system, gross motor function measure 66, motor activities

## Abstract

The aim was to explore longitudinal motor development in children with cerebral palsy (CP) in Sweden with respect to the Gross Motor Function Classification System (GMFCS). In this national CP registry-based study, 2138 children aged 0.5–19 years participated (42% girls). The distribution with respect to GMFCS was I: 49%, II: 16%, III: 10%, IV: 14%, and V: 11%. In total, 5538 assessments (mean 2.7, min–max: 1–9) with the Gross Motor Function Measure-66 were included. Data were analysed using non-linear mixed-effects regression models, and the Stable Limit Model was selected to fit data. Five distinct curves of predicted gross motor development with respect to GMFCS levels were obtained. The achieved motor development was maintained over time. The estimated average GMFM-66 limit and the average age when 90% of the expected limits were reached were at GMFCS I: 88 at age 4.5; GMFCS II: 71 at age 4.2; GMFCS III: 54 at age 3.1; GMFCS IV: 38 at age 2.6, and at GMFCS V: 18 at age 0.9. In conclusion, this is the first national population-based study following motor development in CP. Five distinct curves reported in previous controlled research studies were confirmed. Our study adds knowledge about motor development captured in children’s everyday context.

## 1. Introduction

Motor development such as rolling, sitting, crawling, and walking are essential for children’s early development and affects many aspects of health [1]. According to the definition from the World Health Organization, WHO, early development refers to the cognitive, physical, language, motor, social, and emotional development in children between 0 and 8 years of age [2]. Motor activities offer children the opportunity to discover, explore, and understand their surroundings and have shown beneficial effects on both motor and cognitive development in preschool children [3]. Accordingly, most parents are aware of the importance of their child’s motor development, and for parents who are informed that their child has cerebral palsy (CP), it raises many questions. The first questions are usually whether the child will be able to walk and how the child will develop. Over many years, researchers have tried to predict motor development and specifically the possibility of achieving independent walking in children with CP [4,5,6].

In CP, the development of movement and posture are always affected and are often accompanied by disturbances in sensation, perception, cognition, communication, and behaviour, by epilepsy, and by secondary musculoskeletal problems [7]. CP is the most common motor disorder in childhood in the Western world, with lifelong consequences attributed to disturbances in the developing foetal or infant brain [7]. CP occurs in almost 2/1000 live births in Sweden [8]. Although the brain damage is permanent, the clinical picture often changes during the child’s development [7]. The condition is heterogeneous and is therefore described by neurological subtypes and classification systems. One of them is the Gross Motor Function Classification System (GMFCS I–V), which is used to classify gross motor activity into five levels, varying from children who run and jump to children who need assistance to change all positions [9,10].

The Gross Motor Function Measure 66 (GMFM-66) has commonly been used to evaluate gross motor capacity in children with CP, both in clinical practice and research [11]. GMFM-66 is the motor assessment that is used to follow motor development in the Swedish national quality register for children with CP (CPUP) [12]. In Sweden, all children with CP are offered to participate in CPUP, and more than 95% of the children are included in the register [13]. The GMFM-66 has previously been used to describe longitudinal motor development, and in some studies, patterns of development were illustrated with respect to each GMFCS level [14,15,16,17]. The results revealed the age when the limit of motor development occurred. In children severely affected, the limit was achieved at an early age, whereas children mildly affected achieved the limit at an older age, and thereby had a possibility of reaching a higher motor capacity [14,15,16,17]. 

The first study describing motor development in children with CP was a Canadian study [14]. They followed a stratified sample of 657 children aged between 1 and 13 years, with CP or neuromotor findings consistent with CP, excluding those with tone reduction interventions [14]. Analysis of a total number of 2632 GMFM-66 assessments was described and illustrated by five distinct motor development curves, the Ontario motor growth curves [14]. To assess whether motor ability declined during adolescence, the Canadian sample was further followed until the age of 21 years, by adding 823 assessments with the GMFM-66, resulting in 3455 assessments [15]. The authors reported a decline in motor capacity in children more severely affected, while children less affected remained stable [15]. Longitudinal development of motor capacity was also investigated in a Dutch sample of 423 children with CP aged 1 to 22 years, comprising 1275 assessments with the GMFM-66 [16]. The authors confirmed the existence of five distinct patterns for motor development by level of severity but found no evidence of a decline at any GMFC level [16]. Almost similar results were reported in an Australian study, also investigating motor capacity over time in a sample of 222 children aged from 18 months to 12 years with CP, encompassing 871 GMFM-66 assessments [17]. From the west Sweden CP register, 317 children aged 1 to 15 years participated in a population-based study evaluating motor development with the old version, the GMFM-88 [10]. Unfortunately, the statistical properties of the two versions GMFM-88 and GMFM-66 differ, so the results from Beckung et al. are not comparable with studies using GMFM-66 [5,18,19]. 

Previous studies have contributed to very important knowledge about motor development in children with CP [5,14,15,16,17]. Despite this, it can still be a huge challenge to answer parents’ questions concerning the motor development of their child. These research studies were carried out in connection to university hospitals with specific inclusion criteria and exclusion of children with for example tone reduction treatment or children with an intellectual disability. Consequently, there is still a lack of knowledge about motor development in children with CP in their everyday context.

The aim of this study was therefore to describe motor development in the Swedish national population of children with CP regardless of additional difficulties or previous treatments. We hypothesised that distinct levels of motor development could be observed with respect to the GMFCS and that the achieved level of motor development was maintained over time in children mildly affected, while a decline could be observed in children severely affected.

## 2. Materials and Methods

### 2.1. Design

The study was a longitudinal registry-based study from the Swedish national cerebral palsy surveillance follow-up program, CPUP.

### 2.2. CPUP

CPUP is the Swedish national quality register including children and adults with CP. In the child version, children are regularly followed from infancy until adulthood according to age and GMFCS level [13,20]. In CPUP, the CP diagnosis and CP subtype in each child are confirmed around the age of four years by neuropaediatricians [12]. The diagnostic criteria for CP developed by the Surveillance of Cerebral Palsy in Europe, SCPE, are used [21]. The CP subtypes are based on the dominating neurological findings and classified into either spastic (unilateral or bilateral), dyskinetic (dystonic or choreo-athetotic), or ataxic [21]. If neurological findings are co-occurring, there is an option to register a mixed form. In Sweden, rehabilitation for children with CP is organized in 21 regions with 58 rehabilitation units across the country. The children are systematically examined by local physiotherapists through standardised assessments included in the CPUP protocol [12,22]. Data are electronically entered into the national database. 

### 2.3. Participants 

Inclusion criteria were children in Sweden with a confirmed diagnosis of CP, participating in CPUP, born between 2000 and 2018, with at least one documented GMFM-66 assessment. All children had reached an age of at least four years at the time when data were extracted from the register. 

The study was approved by the Medical Research Ethics Committee at Lund University (23 October 1999 LU 433/99, and 1 March 2011), and permission was obtained to extract data from CPUP (approval date 26 February 2020). Parents and children consented to contribute to research based on reported data. 

### 2.4. Classifications and Assessments

The Gross Motor Function Classification System (GMFCS) was used to classify the performance of gross motor activity [9,10]. The focus of the GMFCS is what the children usually do in their everyday environment. The GMFCS is divided into five levels (GMFCS I–V), spanning from children at GMFCS I who walk without restrictions to children at GMFCS V who need complete assistance with all mobility and are transported in wheelchairs [9]. To accommodate the changing performance at different ages, the system describes five age intervals (0–2, 2–4, 4–6, 6–12 years), and today the expanded and revised version GMFCS-E&R exists, which also adds the age span 12 to 18 years [10]. In the present study, the name GMFCS will be used hereinafter. The GMFCS has shown good psychometric properties [22]. The GMFCS level registered close to four years of age was used to ensure a confirmed CP subtype [23]. The GMFCS level at the entrance of CPUP was used for children included at an older age. 

The GMFM-66 is a standardised observational measure evaluating change in gross motor capacity in children with CP [11]. The measure includes 66 items related to lying, rolling, sitting, crawling, kneeling, standing, walking, running, and jumping. In the test situation, the physiotherapist encourages the children to show their maximum capacity. The most difficult items correspond to what children with typical development can do when they are five years old. Each item is scored on a four-point Likert scale (0–3) according to the manual, where each item and each score are defined. The test is Rasch analysed and to obtain a total GMFM-66 score, the Gross Motor Ability Estimator (GMAE-2) Scoring Software is used [24]. The items are organised in increasing difficulty from 0 (low capacity) to 100 (high capacity) along an interval scale. The GMFM-66 has shown good psychometric properties and is sensitive to change in children with CP [24,25]. In Sweden, the GMFM-66 was introduced almost 40 years ago and is used both in clinical practice and in research today. In order to achieve high-quality data in the national quality register (CPUP), profession-specific education and training are carried out based on the needs reported by the responsible coordinator in each region. Education on the GMFM-66 takes place continuously to train new colleagues and maintain competence among experienced physiotherapists both at the local habilitation units and on a central level. Registering the GMFM-66 score in CPUP has been optional since 8 December 2006. In the present study, the assessments were performed by physiotherapists at the local rehabilitation units, and they also registered the results in the national register.

### 2.5. Procedure

Data were extracted from the CPUP database concerning birthdate, sex, CP subtype, GMFCS, and dates and scores of GMFM-66 assessments, recorded during the period 8 December 2006–12 April 2022. At the time when data were extracted, all children had reached an age of at least four years, to ensure a confirmed CP diagnosis and a reliable GMFCS classification. Therefore, this information was used throughout the longitudinal analysis and data from children born between 2000 and 2018 were included.

### 2.6. Data and Statistical Analysis

The data are presented as numbers and percentages for age group, sex, subtype of CP, and GMFCS. Scores from GMFM-66 are presented as means, 95% confidence intervals (CI), and standard deviations (SD) with respect to GMFCS level and age group. Age was categorized into five groups according to the following half-open age intervals (lower bound not included): 0–2, 2–6, 6–9, 9–12, and 12–19 years. Each age group can include multiple observations from the same child and the same child can be part of multiple age groups. 

The GMFM-66 was modelled as a non-linear function of age using two different mixed effects models for longitudinal analysis. The two models were compared to find estimates of the average pattern of GMFM-66 change over age for each GMFCS level. Following the procedure in a previous study by Hanna et al. [15], the model fitting and comparisons were conducted for each GMFCS level separately [15]. The first model was the so-called Stable Limit model, which assumes a rapid increase in young age until a plateau is reached where the GMFM-66 stabilizes. Consequently, the Stable Limit model has two parameters, namely the upper estimated limit of the GMFM-66 and the increase rate. The rate parameter was transformed to Age_90_, which is the average age when children reach 90% of their expected GMFM-66 limit [15]. The second model was the so-called Peak and Decline model, assuming a rapid increase at a young age until the age when the highest value is obtained, the peak, followed by a slower decrease in GMFM-66. The Peak and Decline model has three parameters, the rate, the predicted GMFM-66 for an arbitrary reference age, and the upper asymptote of GMFM-66. Individual variation was accounted for by adding a random effect to each parameter. An unstructured variance–covariance structure was used for the random effects. All parameters were estimated using maximum likelihood estimation. To facilitate convergence, initial estimates were specified for the fixed effects (Supplementary Materials: Table S1 or for more details on the models and their implementation, see Hanna et al., 2008 [15]). Goodness-of-fit was evaluated based on the Akaike Information Criterion (AIC) statistic. All analyses were conducted using STATA version 16.1 (StataCorp, College Station, TX, USA).

## 3. Results

At the time when data were extracted, 2138 children were registered in CPUP and fulfilled the inclusion criteria. Of the total population, 42 percent were girls, 58 percent were boys, and data on sex were missing in 0.3 percent. All neurological CP subtypes and GMFCS levels were represented (Table 1). 

At the time of the first GMFM-66 assessment, the mean age of the total group was 5.7 (SD 3.7) years, ranging from 0.5 to 19 years of age. The mean age at the first GMFM-66 assessment across age groups and the number of children at each GMFCS level are presented in Table 2.

In total, 5538 GMFM-66 assessments were performed. The mean number of assessments per child was 2.7 with a variation from 1 to 9 assessments (Table 1). The mean number of assessments at GMFCS level I was 2.6, 2.9 at GMFCS II, 2.8 at GMFCS III, 2.6 at GMFCS IV, and 2.0 at GMFCS V (Table S1). The longitudinal mean GMFM-66 scores by each GMFCS level and age group are presented in Table 3. 

The Stable Limit Model was used since convergence was not achieved in any GMFCS level with the Peak and Decline model. In addition, better AIC values were found for the Stable Limit model (Table S1). The lengths of the confidence intervals were small and indicated a pronounced difference between each GMFCS level (Table S1). At GMFCS I, the estimated GMFM-66 limit was 88.1 (87.3–88.9) and the age at which the children were expected to achieve 90% (Age_90_) was 4.5 years; at GMFCS II, the estimated GMFM-66 limit was 71.3 (70.2–72.4) and the Age_90_ was 4.2 years; at GMFCS III, the estimated GMFM-66 limit was 54.3 (53.3–55.3) and Age_90_ was 3.1 years; at GMFCS IV, the estimated GMFM-66 limit was 38.5 (37.5–39.5) and Age_90_ was 2.6 years, and at GMFCS V, the estimated GMFM-66 limit was 17.9 (16.8–19.1) and Age_90_ was 0.9 years (Supplementary Materials: Table S1). 

**Figure 1:**
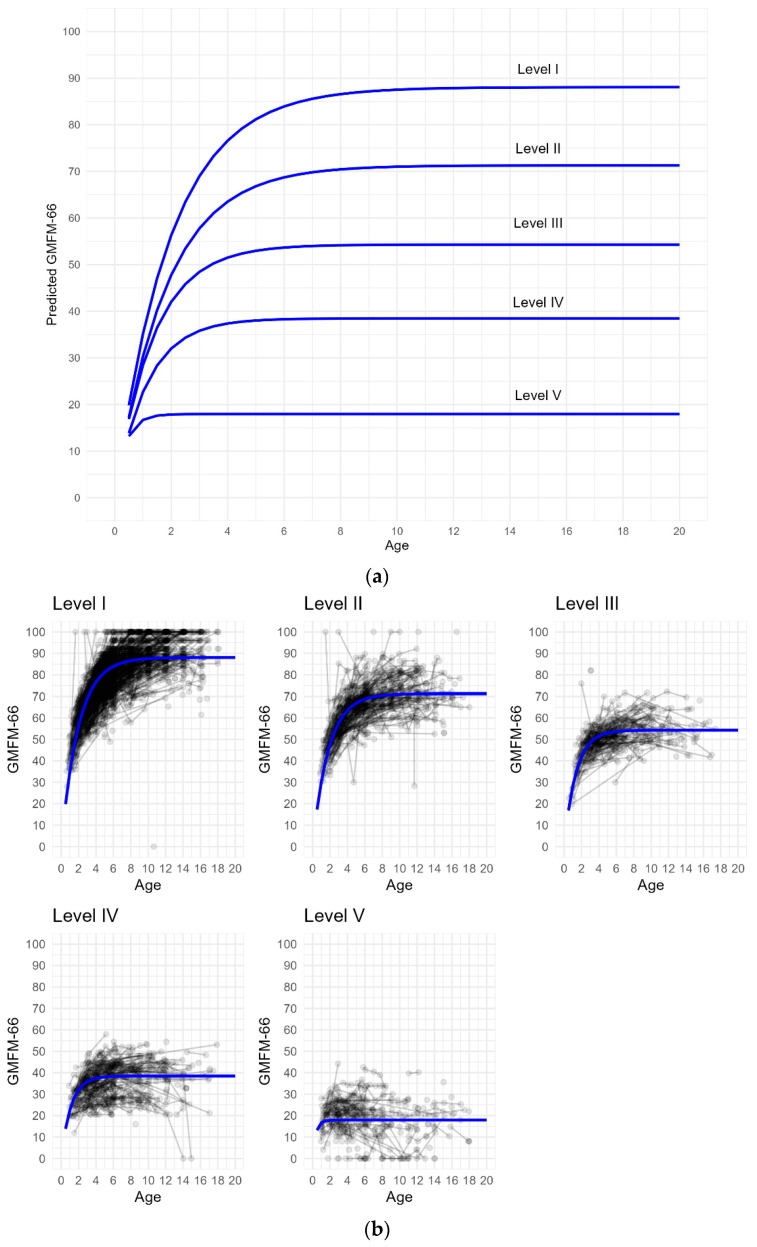
Illustrations of gross motor development. Results from the Gross Motor Function Measure (GMFM-66) by age and with respect to Gross Motor Function Classification System (GMFCS) level I, II, III, IV, and V. (**a**) Predicted average GMFM-66 scores. (**b**) Observed and predicted GMFM-66 scores. The curved blue lines indicate predicted average GMFM-66 scores. The black circles indicate observed GMFM-66 scores.

Five distinct curves of predicted gross motor development with respect to GMFCS levels were observed (Figure 1a). The predicted average patterns of the GMFM-66 change over age for each GMFCS level are illustrated in Figure 1a,b. The longitudinal trend in predicted GMFM-66 scores by GMFCS level indicated no obvious decline. 

## 4. Discussion

This is the first national population-based register study describing motor development in children aged six months to 19 years with CP. Our results confirmed the occurrence of five distinct levels of motor development with respect to the GMFCS, as previously reported [14,15,16,17]. Furthermore, the results revealed that the predicted GMFM-66 limit was slightly lower and that children reached the predicted limit at a younger age in some GMFCS levels, compared to previously reported results. The hypothesis was confirmed regarding the maintenance of achieved motor development in children mildly affected, in contrast to the fact that a decline was not discovered by the statistical method used in children severely affected, although a trend of declines could be observed. 

In our study, the Stable Limit model was used since the model showed a better fit based on AIC statistics in comparison to the Peak and Decline model. Corresponding results were described in the Dutch study, where the Stable Limit model also was used based on a better fit from AIC statistics [16]. However, our results from the Peak and Decline model need to be regarded with caution since convergence was not achieved at any GMFCS level. 

Our results indicated that children in the Swedish population achieved 90 percent of their estimated GMFM-66 limit at a younger age in comparison to what has been described previously [14,15,16]. In addition, the estimated GMFM-66 limit was somewhat lower in comparison to the Dutch study at all GMFCS levels [16], and to some extent also lower at GMFCS I, IV, and V in comparison to the Canadian study [15]. One possible explanation for the differences in our study could be that all children with CP were included regardless of whether they had an intellectual disability, had additional diseases and disorders, or received tone reduction treatment. Associations between intellectual disability and motor activity have previously been reported [26,27]. A further explanation for the lower GMFM-66 limit could be that interventions such as orthotics, assistive devices, and adaptations of the environment are free of charge and are commonly introduced early in Swedish children’s lives. The children mainly train towards individual tailored goals in their everyday environment where these adaptations exist [28]. In the present study, motor development was assessed with the GMFM-66, which captures the children’s motor capacity without access to orthosis, assistive devices, or adaptations. Many children were therefore not used to acting without assistive devices and orthoses, which might have negatively influenced their capacity. The impact of orthoses on gait and gross motor function was described in a meta-analysis and showed positive results [29]. Our results possibly could have been higher with the use of the children’s habitual devices. However, the results of the present study only revealed the development of motor capacity and not how the children performed in their everyday environment. 

The aim of rehabilitation in Sweden is to offer children with CP to participate in everyday life; this includes independent mobility giving them the opportunity to discover, explore, and understand their environment. To assess motor performance in children’s everyday environment, the Pediatric Evaluation of Disability Inventory (PEDI) was used in an Australian study [17]. Interesting results were presented, where mobility continued to develop in the children’s everyday environment, while a plateau of motor capacity as assessed by the GMFM-66 was observed in children at GMFCS II and GMFCS III [17]. The authors concluded that factors such as assistive devices and supportive environments were important for children’s mobility in everyday settings. However, despite this, a strong positive association between the GMFM-66 and the PEDI mobility domain was reported [17].

The present study entails all children from north to south of Sweden, comprising 21 regions with a total of 58 local rehabilitation units. The assessments were carried out by the local physiotherapists at each unit in their everyday clinical work where follow-ups with CPUP were performed. Accordingly, all children with CP were included. In previous research studies, a selected sample of children was included, and the data collection took place in connection with university hospitals. In the early Canadian study, children were excluded if they received any tone reduction interventions [14]. In the Dutch study, children with additional diseases and disorders other than CP were excluded, and furthermore, children aged 16–24 years with intellectual disability were excluded [16]. 

In the present study, observations of the mean values from 5538 GMFM-66 scores of 2138 children revealed the occurrence of five distinct patterns of motor development with respect to each GMFCS level over age. The lengths of the confidence intervals were small and indicated a pronounced difference between each GMFCS level. Corresponding results have been described earlier [14,15,16,17]. In the present study, we observed motor progress with increasing age in children at GMFCS I and a maintenance of the achieved motor capacity during the follow-up period. In contrast, motor development in children at GMFCS V did not continue after the first two years. Similar results have been described previously in children at GMFCS I and V [14,15,16,17].

The representation of participants in our study, with respect to sex, neurological subtypes, and GMFCS levels, was in accordance with a previously reported study from the Swedish national population [13]. In the current study, the representation of boys compared to girls was higher but almost in agreement with what has been previously reported in studies describing motor development in CP [15,16]. In our population, the representation of children with dyskinetic CP was higher in comparison to the Canadian and Dutch studies, the representation of children with spastic CP was almost similar, while the representation of mixed form was lower [14,15,16]. With respect to GMFCS levels, the proportion of children represented in our study was almost like the Dutch and the Australian studies [16,17]. In these studies, the proportion of children at GMFCS I was high, whereas it was lower at GMFCS III, IV, and V. In the Canadian study, a reversed representation was present with a high proportion of children at GMFCS III, IV, and V [14,15]. 

The total number of included children in the present study was higher than previously reported in studies exploring motor development in CP [14,15,16,17]. At inclusion, our population was somewhat younger compared to the Canadian and the Dutch studies, and we followed them to the age of 19 years, whereas they followed their participants to the age of 21 and 22 years, respectively [14,16]. In addition, the number of assessments with the GMFM-66 per child was lower in the present study in comparison to the Canadian study and slightly lower compared to the Dutch study [15,16]. In the present study, the mean number of assessments per child was 2.7 compared to the Dutch study with a mean of 3.1 assessments and to the Canadian study with a median number of 5 assessments per child [15,16]. In summary, the most obvious differences between these studies concerned study design, the total number of children, the total number of assessments per child, and the representation of children with respect to the GMFCS level [14,15,16,17]. The high number of assessments per child, in combination with the high proportion of children severely affected in the Canadian study, possibly enabled them to capture a decline in children severely affected by using the statistical model Peak and Decline [15]. 

### Strengths and Limitations

Reporting results from a national register study could entail both strengths and limitations. One limitation is that the participants have not undergone a strict selection, and the results can be distorted by various confounders. However, since the aim was to follow the natural motor development and thereby reflect the clinical reality, children with CP in Sweden were included regardless of additional disorders. A further limitation could be the lack of control over data collection since many different physiotherapists throughout the country of Sweden examined the children and recorded the data. Despite this, the results are in line with previous controlled research studies [14,15,16,17]. In addition, the high validity and high reliability of assessments with the GMFM-66 and the stability of classification with GMFCS have been reported in previous studies [19,24]. Including children who only had one assessment could be considered a limitation, but this could also be a strength to reduce the risk of inclusion bias. The inclusion of 2138 children could be considered a strength in comparison to previous studies where the number varied from 222 to 657 children [5,14,15,16,17]. The decision to include children with at least one GMFM-66 assessment was in line with the overall intention of CPUP, which is to follow all children with CP regardless of environmental factors such as long distances and/or social background. Another strength is that the representation of participants is in accordance with previously reported studies from the Swedish national population with respect to sex, neurological subtypes, and GMFCS levels [13]. An additional strength was that the results are based on a national population of children who were examined in their everyday context. Together with results from previous controlled research studies, the new knowledge contributes to answering parents’ questions about motor development in their children.

## 5. Conclusions

This is the first national study following motor development over time in children with CP. The results confirm the presence of five distinct curves in relation to severity, as previously reported. The achieved level of motor development was maintained by age. However, the predicted GMFM-66 limit was slightly lower, and children reached 90% of the predicted limit at a younger age in some GMFCS levels compared to previously reported results. Children were included regardless of intellectual disability, additional disorders, and previous treatments. This study provides new information on motor developmental trajectories as a function of GMFCS levels, captured in the everyday context in a national population. Future studies on the impact of various factors related to motor development such as CP subtypes, accompanying disorders and interventions would contribute to improving the prediction of longitudinal motor development.

## Figures and Tables

**Table 1:** Descriptive characteristics of 2138 participants with cerebral palsy and the GMFM-66 assessments.

**Sex**	**n ^1^ (% ^2^)**
Female/male/missing	887 (42)/1245 (58)/6 (0.3)
**Neurological subtype**	**n ^1^ (% ^2^)**
Spastic unilateral	664 (31)
Spastic bilateral	732 (34)
Spastic not specified	293 (13.7)
Dyskinetic	216 (10)
Ataxic	81 (3.8)
Mixed form	72 (3.4)
Not specified	80 (3.7)
**GMFCS ^3^**	**n ^1^ (% ^2^)**
GMFCS I	1060 (49)
GMFCS II	346 (16)
GMFCS III	209 (10)
GMFCS IV	292 (14)
GMFCS V	231 (11)
**Number of GMFM-66 ^4^**	**n^1^ (% ^2^)**
1/2/3/	900 (42)/489 (23)/269 (13)
4/5/6	151 (7)/104 (5)/68 (3)
7/8/9	34 (2)/24 (1)/99 (5)

n ^1^ = number of children, % ^2^ = percent, GMFCS ^3^ = Gross Motor Function Classification System, GMFM-66 ^4^ = Gross Motor Function Measure 66.

**Table 2:** Representation of children by Gross Motor Function Classification System (GMFCS) and age group at the first Gross Motor Function Measure (GMFM-66).

Age Group (y ^1^)	0–2	2–6	6–9	9–12	12–19	Total
GMFCS I (n ^2^)	96	589	174	101	100	1060
Mean (y ^1^)	1.47	3.86	7.25	10.28	14.07	5.78
(SD)	(0.35)	(1.13)	(0.93)	(0.88)	(1.66)	(3.70)
GMFCS II (n ^2^)	50	164	64	37	31	346
Mean	1.51	3.76	7.10	9.92	13.96	5.63
(SD)	(0.32)	(1.13)	(0.81)	(0.79)	(1.50)	(3.71)
GMFCS III (n ^2^)	29	110	39	14	17	209
Mean	1.41	3.60	7.21	10.51	13.85	5.26
(SD)	(0.37)	(1.15)	(1.00)	(0.95)	(1.29)	(3.63)
GMFCS IV (n ^2^)	44	143	50	36	19	292
Mean	1.50	3.76	7.31	10.34	13.54	5.48
(SD)	(0.30)	(1.13)	(1.03)	(0.96)	(1.56)	(3.56)
GMFCS V (n ^2^)	37	94	40	28	32	231
Mean	1.41	3.97	7.17	10.51	14.22	6.32
(SD)	(0.32)	(1.16)	(0.93)	(0.86)	(1.79)	(4.28)

y ^1^ = year, n ^2^ = number.

**Table 3:** Results from Gross Motor Function Measure (GMFM-66) by age group and Gross Motor Function Classification System (GMFCS) level I, II, III, IV, and V.

Age Group (y ^1^)		0–2	2–6	6–9	9–12	12–19
	GMFM-66	n ^b^ = 340	n ^b^ = 2.840	n ^b^ = 1.157	n ^b^ = 666	n ^b^ = 535
GMFCS I n ^a^ = 1060	Mean 95%CI SD n ^c^	52.2 50.53–53.94 9.9 131	73.1 72.59–73.64 10.0 1424	85.3 84.56–86.04 9.0 566	90.3 89.26–91.34 9.5 321	91.4 90.43–92.29 8.1 294
GMFCS II n ^a^ = 346	Mean 95%CI SD n ^c^	46.1 43.67–48.43 9.8 67	61.3 60.51–62.16 9.1 468	70.0 68.90–71.13 8.4 221	72.5 70.96–74.04 9.2 140	70.8 68.96–72.60 8.9 94
GMFCS III n ^a^ = 209	Mean 95%CI SD n ^c^	37.1 33.80–40.39 10.0 38	50.0 49.30–50.63 6.1 327	54.3 53.06- 55.52 6.8 119	56.1 54.38–57.79 6.8 63	53.6 51.40–55.76 7.0 42
GMFCS IV n ^a^ = 292	Mean 95%CI SD n ^c^	27.1 25.53–28.74 5.8 53	34.9 34.14–35.57 7.3 399	38.0 36.74–39.21 7.9 160	38.2 36.51–39.90 8.3 93	35.8 32.96–38.69 10.7 56
GMFCS V n ^a^ = 231	Mean 95%CI SD n ^c^	18.9 17.13–20.70 6.3 51	20.7 19.64–21.69 7.7 222	18.3 16.28–20.37 9.8 91	15.3 12.26–18.43 10.7 49	17.4 14.81–19.92 8.9 49

y ^1^= year, n ^a^ = total number of children within each GMFCS level across age groups. n ^b^ = total number of GMFM-66 assessments including multiple assessments per child within age groups. n ^c^ = number of assessments including multiple assessments per child within and across age groups and GMFCS levels.

## Data Availability

The CPUP database is owned by Skane Regional Council (Region Skane, 29 189 Kristianstad, Sweden) and the NPR database by the National Board of Health and Welfare (Socialstyrelsen, 10,630 Stockholm, Sweden). The datasets used and analysed during the current study are available from the corresponding author on reasonable request and with permission of Skane Regional Council and the National Board of Health and Welfare.

## Supplementary Materials

The following supporting information can be downloaded at: https://www.mdpi.com/article/10.3390/children10121864/s1, Table S1: Comparisons of two models for longitudinal analysis.

## Abbreviations

AIC	Akaike Information Criterion (AIC)
CI	Confidence Interval
CP	Cerebral Palsy
CPUP	Cerebral Palsy Surveillance Follow-Up Program
GMFCS	Gross Motor Function Classification System
GMFM-66	Gross Motor Function Measure 66
PEDI	Pediatric Evaluation of Disability Inventory
SD	Standard Deviation
