# Supplementary material for: Motor Development in Children with Cerebral Palsy in Sweden—A Population-Based Longitudinal Register Study
Source: Children (Basel). 2023 Nov 28;10(12):1864. doi: 10.3390/children10121864 (PMC10741609; doi:10.3390/children10121864)
Supplement: Supplementary file 1 [file children-10-01864-s001.zip › children-2728496-supplementary.pdf]

Table S1. Comparisons of two models for longitudinal analysis.
Comparison of Akaike Information Criterion (AIC) between Stable Limit (SL) model and Peak and Decline (PD) model and presentation of SL model with respect to Gross Motor Function Classification System (GMFCS).

	**GMFCS**	**I** n[a]=1060	**II** n[a]=346	**III** n[a]=209	**IV** n[a]=292	**V** n[a]=231
	Mean n observations/child	2,6	2,9	2,8	2,6	2,0
AIC	SL model	18198*	6585*	3534*	4714*	3040*
	PD model	19457	7202	3783	5300	3729
SL model	GMFM-66 limit (95% CI)	88,1 (87,3–88,9)	71,3 (70,2–72,4)	54,3 (53,3–55,3)	38,5 (37,5–39,5)	17,9 (16,8–19,1)
	Age^{90} (95% CI)	4,5 (4,4–4,6)	4,2 (3,9–4,4)	3.1 (2,9–3,3)	2,6 (2,4–2,8)	0,9 (0,7–1,0)
	Residual SD	4,0	4,6	2,8	3,2	3,9

n[a] = Total number of children
*= Lower AIC for SL model in comparison to PD model which gives SL model better fit
Age^{90}= The average age when children reach 90% of their expected GMFM-66 limit
